# Xpert MTB/RIF Ultra Trace Results: Decision Support for the Treatment of Extrapulmonary Tuberculosis in Low TB Burden Countries

**DOI:** 10.3390/jcm12093148

**Published:** 2023-04-27

**Authors:** Aurélie Guillouzouic, Alice Gaudart, Eve Tessier, Karine Risso, Farida Hamdad, Corentine Alauzet, Pierre Vaillant, Christelle Koebel, Loïc Kassegne, Rachel Chenouard, Pierre Abgueguen, Cécile Le Brun, Simon Jamard, Raphaël Lecomte, Maeva Lefebvre, Pascale Bémer

**Affiliations:** 1 Department of Microbiology, Nantes University Hospital, 44093 Nantes, France; 2 Department of Microbiology, Nice University Hospital, 06000 Nice, France; 3 Department of Infectious Diseases, Nice University Hospital, 06000 Nice, France; 4 Department of Microbiology, Nancy University Hospital, 54035 Nancy, France; 5 Pulmonary Department, Nancy University Hospital, 54035 Nancy, France; 6 Department of Microbiology, Strasbourg University Hospital, 67091 Strasbourg, France; 7 Pulmonary Department, Strasbourg University Hospital, 67091 Strasbourg, France; 8 Department of Microbiology, Angers University Hospital, 49000 Angers, France; 9 Department of Infectious Diseases, Angers University Hospital, 49000 Angers, France; 10 Department of Microbiology, Tours University Hospital, 37081 Tours, France; 11 Department of Infectious Diseases, Tours University Hospital, 37081 Tours, France; 12 Department of Infectious Diseases, Nantes University Hospital, 44095 Nantes, France

**Keywords:** extrapulmonary tuberculosis, Xpert MTB/RIF Ultra test, trace result, clinician’s confidence, argument to treat

## Abstract

Objectives. Extrapulmonary tuberculosis (EPTB) can be difficult to diagnose, especially in severe forms. The Xpert MTB/RIF Ultra test introduced an additional category called trace to reference very small amounts of *Mycobacterium tuberculosis* complex (MTBC) DNA. The objective of our multicenter study was to evaluate whether the trace result on an extrapulmonary (EP) sample is a sufficient argument to consider diagnosing tuberculosis and starting treatment, even in severe cases. Methods. A retrospective, multicenter cohort study was conducted from 2018 to 2022. Patients strongly suspected of EPTB with a trace result on an EP specimen were included. Hospital records were reviewed for clinical, treatment, and paraclinical data. Results. A total of 52 patients were included, with a severe form in 22/52 (42.3%) cases. Culture was positive for MTBC in 33/46 (71.7%) cases. Histological analysis showed granulomas in 36/45 (80.0%) cases. An Ultra trace result with a presumptive diagnosis of TB led to the decision to treat 41/52 (78.8%) patients. All patients were started on first-line anti-TB therapy (median duration of 6.1 months), with a favorable outcome in 31/35 (88.6%) patients. The presence of a small amount of MTBC genome in EPTB is a sufficient argument to treat patients across a large region of France.

## 1. Introduction

Extrapulmonary tuberculosis (EPTB) accounts for 16% of reported active TB cases worldwide in 2019 [1], with a higher proportion in children and HIV-positive individuals. The most common sites of involvement are the pleura and lymph nodes. Neuromeningeal, digestive, and disseminated forms, although less common, are nonetheless extremely severe and difficult to diagnose, and are therefore more likely to result in a fatal outcome [2,3]. Compared with pulmonary tuberculosis, extrapulmonary forms are characterized by less suggestive symptoms, are more difficult to diagnose, have paucibacillary specimens, and more frequently require invasive procedures to obtain clinical samples for culture. The diagnosis of EPTB still faces challenges in clinical practice [4]. The presumptive diagnosis most often combines clinical, radiological, and/or histological criteria.

The Xpert MTB/RIF Ultra test, a real-time PCR test that recently replaced the Xpert MTB/RIF test, lowers the detection limit for *Mycobacterium tuberculosis* complex (MTBC). Detection of bacillary load, based on IS*1081*-IS*6110* amplification, is categorized as high, medium, low, very low, and trace-positive results. The additional category called trace is caused by very small amounts of bacterial DNA and cannot provide results for rifampicin (RIF) susceptibility as the number of IS copies is higher than *rpoB* (monocopy in the genome of MTBC) [5]. While the introduction of this semi-quantitative trace category undoubtedly improves sensitivity (particularly in smear negative samples) upon the previous version of Xpert, it may decrease specificity; therefore, the clinical management of patients with trace results remains challenging.

Dowling et al. [6] reported that the prevalence of trace results varies highly, from 3 to 55%, based on the series. The WHO Technical Expert Group (TEG) developed criteria for the clinical interpretation of trace results in 2017 [7]. The goal was to limit the potential overtreatment of patients with false-positive results while detecting more patients with TB. The TEG recommended that TB treatment should be initiated for the following high-risk patients with trace results: people living with human immune-deficiency virus, children, and patients with EPTB [7].

Several studies have evaluated the clinical impact of Ultra trace results. The first study specifically devoted to the analysis of trace results involved 21 laboratories with 317 (11.9%) tests with trace results among 2659 positive Ultra tests [8]. Samples of pulmonary origin represented 68% of the total. About 25% of trace results were considered to be false positives due to a history of treatment or an unlikely or excluded diagnosis of TB [8].

The main objective of Dowling’s study was to identify predictive factors of active TB, defined as a positive culture for *M. tuberculosis*, in patients with a trace result [6]. More positive TB cultures were significantly observed in patients with EPTB samples than pulmonary TB samples. Three major criteria were identified as predictive tools for trace results: EPTB, TB symptoms, and no previous TB disease, especially in high-burden TB settings [6]. These three predictive factors can help clinicians diagnose patients with a trace result and should be included in TB diagnostic algorithms to interpret the trace result, particularly in settings with a high incidence of TB.

Finally, a very recent monocentric study evaluated clinical management of trace results in low TB burden settings [9]. Of the 59 patients included in the analysis, 34 had nonrespiratory samples with 55.9% of lymph node biopsies. All the 34 patients had either symptoms and/or radiological findings consistent with TB, meeting the definition of presumptive TB. A total of 57 (96.6%) patients started anti-TB treatment, based on clinical and/or radiological findings and an Ultra trace result with a delay of 4 ± 4.7 days after the trace result [9].

The main objective of our multicenter study was to evaluate whether an Ultra trace result on an extrapulmonary specimen associated with a presumptive diagnosis of EPTB led clinicians to initiate anti-TB treatment before the culture result, including the most severe EPTB forms such as digestive, neuromeningeal, and disseminated tuberculosis. Secondary objectives were the correlation of trace results with culture and histological findings.

## 2. Materials and Methods

### 2.1. Study Design

A retrospective multicenter cohort study examining patients with EPTB was conducted in six French hospitals (Angers, Nancy, Nantes, Nice, Strasbourg, and Tours). The study protocol was approved by the ethics review boards in each center, with the University of Nantes as the coordinating center. Patient consent was waived because of the retrospective design of the study, and there was no study-related intervention.

### 2.2. Study Population

From January 2018 to December 2022, the mycobacterial database of each center was retrospectively analyzed to identify adult and pediatric cases with an Xpert trace result on an extrapulmonary specimen in the setting of high clinical and radiological suspicion of extrapulmonary TB. Patients with a trace result on their pulmonary sample or without suspicion of TB (i.e., diagnosis other than TB and no clinical symptoms and/or radiological signs of presumptive TB), with a prior TB treatment (<5 years), or currently undergoing TB treatment were excluded.

Disseminated TB was defined as isolation of *M. tuberculosis* from blood, bone marrow, liver biopsy specimen, or from at least two noncontiguous organs, or isolation of *M. tuberculosis* from one organ and histologic demonstration of TB from the bone marrow, liver biopsy, or another noncontiguous organ [2,3].

Disseminated, digestive or meningeal TB in adults and other forms than lymphadenopathy in children were considered severe EPTB.

### 2.3. Data Collection

The available hospital records were reviewed for demographic data, history of TB treatment, human immunodeficiency virus (HIV) status, definitive diagnosis, EPTB anatomical sites, and the start date and duration of anti-TB treatment. Paper and electronic records were accessed with appropriate permission.

The following laboratory results were collected: histological analysis, acid-fast bacilli smear results, time to culture positivity, *Mycobacterium tuberculosis* complex (MTBC) species identification, and antibiotic susceptibility. The mean time between the Xpert trace result and the positive culture for MTBC was calculated. Time between the Xpert trace result and the start of TB treatment was calculated.

The outcome was considered favorable if the clinical and imaging findings were compatible with cure, and as unfavorable if the clinical or radiological findings were consistent with active TB, and/or if at least one extrapulmonary sample was positive for culture of MTC at the end of the treatment.

Data were anonymized and recorded in Microsoft Excel 2010 (Microsoft, Redmond, WA, USA).

### 2.4. Microbiological Methods

Xpert MTB/RIF Ultra assay (Ultra, Cepheid, Sunnyvale, CA, USA) was performed according to the manufacturer’s instructions.

Briefly, biopsy specimens were mechanically homogenized and resuspended in saline or in phosphate buffer.

Fluid or homogenized tissues samples were pretreated with the manufacturer’s sample reagent for 15 min with occasional shaking and then added in the sample chamber of the cartridge for automatic processing. The system automatically interpreted the fluorescent signal of IS*6110*–IS*1081* and *rpoB* probes according to an internal algorithm to detect MTBC genome and rifampicin resistance, respectively. Positive samples results were classified into semiquantitative categories (i.e., high, medium, low, and very low) with or without detected rifampicin resistance. An additional category corresponding to trace result identified samples with the lowest amount of MTBC DNA with indeterminate rifampicin susceptibility (presence of IS*6110* and/or IS*1081* molecular signal with a cycle threshold ≤37 and absence of molecular signal from at least 3 *rpoB* probes).

For all specimens, microscopic examination was performed by Ziehl–Neelsen and/or auramin staining. Then, specimens were inoculated onto a liquid culture medium (mainly BD BACTEC MGIT 960 system, Becton Dickinson, Le Pont De Claix, France) and a solid culture medium (mainly Löwenstein–Jensen) and incubated for at least 42 days. The identification of MTBC at the species level was based on molecular commercial kits (GenoType MTBC; Hain Lifescience, Nehren, Germany). Susceptibility of MTBC isolates to first-line drugs (Isoniazid (INH), Rifampicin, Ethambutol, Pyrazinamide) using MGIT 960 system (BD BACTEC™ MGIT™ AST SIRE, BD Sparks, Sparks Glencoe, MD, USA) was performed as recommended.

Microscopic examination and cultures were not performed for samples embedded in paraffin (n = 6).

## 3. Results

**Figure 1:**
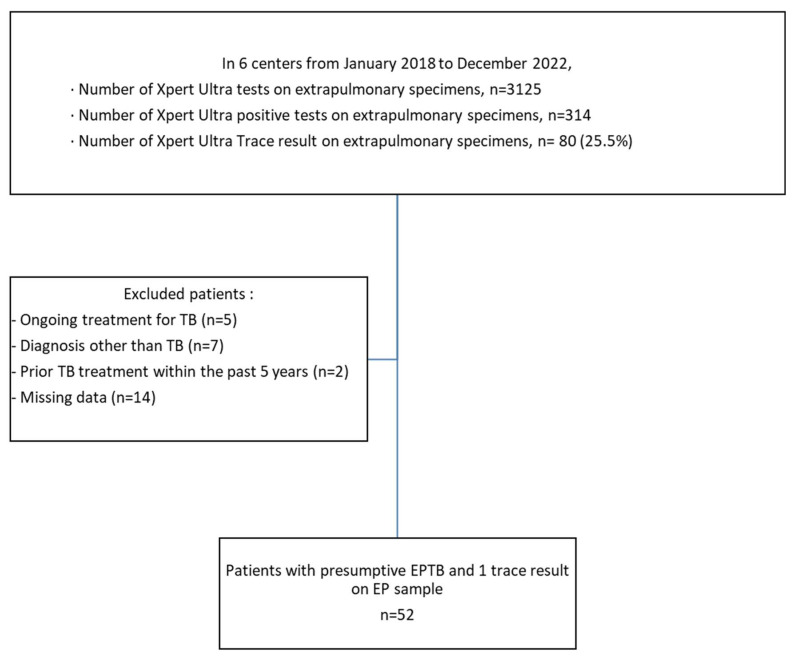
Flow-chart for the study. EP: extrapulmonary.

Over the 5-year study period, 80 patients had a trace result on an extrapulmonary sample. The following patients were excluded from the study: 5 patients being treated for TB, 7 patients with a diagnosis other than TB, 2 patients treated for TB in the last 5 years, and 14 patients with missing data (see Figure 1).

A total of 52 patients with presumptive EPTB and trace result on an EP sample were included (Nantes n = 12, Nice n = 10, Nancy n = 10, Strasbourg n = 9, Angers n = 7, Tours n = 4) (Figure 1).

### 3.1. Characteristics of the Study Population

The median age was 30 years, including 4 patients under 15 years (Table 1). Most of patients (38/52, 73.1%) originated from high-incidence TB countries. Two patients had been treated for TB 12 and 19 years ago, and 2 were HIV positive.

A total of 4 of the 52 patients (7.7%) were pediatric patients (1-year-old, 3-year-old, and 2 15-year-olds), all of them belonged to families originating from high TB burden countries. Two of them had a severe form of EPTB (digestive n = 1, neuromeningeal n = 1). Among the 48 adult patients, 20 (41.7%) had a particularly severe form of EPTB (disseminated n = 14, digestive n = 3, neuromeningeal n = 3) (Table 1). A concomitant pulmonary TB was present in 16/52 patients (30.7%).

### 3.2. Clinical and/or Radiological Findings

Symptoms suggestive of TB and/or radiological findings consistent with TB were present in all patients, consistent with the diagnosis of presumptive EPTB.

### 3.3. Histological and Microbiological Results

Among the disseminated forms, the trace result was obtained from lymph node (n = 4), peritoneal tissue (n = 4), bone tissue (n = 2), heart tissue (n = 1), hepatic tissue (n = 1), cerebrospinal fluid (n = 1), and cutaneous tissue (n = 1).

All trace samples were smear negative, and 33/46 (71.7%) samples were positive in culture for Mycobacterium tuberculosis complex after a mean incubation time of 18.9 days. The time to culture positivity were longer for bone and cardiac tissue (Table 2).

The culture of 13/46 samples remained sterile (lymph node n = 4, pleural fluid n = 4, vertebral n = 1, peritoneal n = 1, cerebrospinal fluid n = 1, epididymis n = 1, cutaneous tissue n = 1) and granulomas were found in 8/12 (66.7%) cases (one histological analysis not realized). Six samples could not be cultured as they were embedded in paraffin.

A 2nd PCR Xpert Ultra was performed for 22 of the 52 patients, on extrapulmonary samples in 14 cases, and pulmonary samples in 8 cases. The PCR was positive in 9/22 (40.9%) cases, in seven cases on an extrapulmonary sample of the same location, and in two cases on a pulmonary sample. The search for rifampicin resistance was possible in six cases, and uninterpretable in three cases because of trace results.

*M. tuberculosis* was the species identified in 28/33 cases (84.8%), *M. africanum* in 3/33 (9.1%), and *M. bovis* in 2/33 (6.1%). The drug susceptibility testing confirmed sensitivity to first line antituberculosis drugs for 31/33 strains (93.9%) including the two M. bovis strains intrinsically resistant to pyrazinamide. Two *M. tuberculosis* strains that were monoresistant to INH were detected. No strain presented a multidrug-resistant (MDR, defined as resistance to INH and RIF) phenotype was found.

Histological analysis was available for 45/52 (86.5%) patients. Granulomas were observed in 36/45 (80%) cases, accompanied by caseous necrosis in 25/45 (55.6%) cases. (Table 3). In a case of chronic diarrhea in a 3-year-old child, the presence of granulomatous lymphadenitis without associated necrosis led to the initial suspicion of sarcoidosis. The trace result of the peritoneal biopsy led to the initiation of antituberculosis treatment; the diagnosis of peritoneal tuberculosis was finally confirmed by the positive culture 18 days later.

### 3.4. Role of the Ultra Trace Result in the Decision to Treat

**Figure 2:**
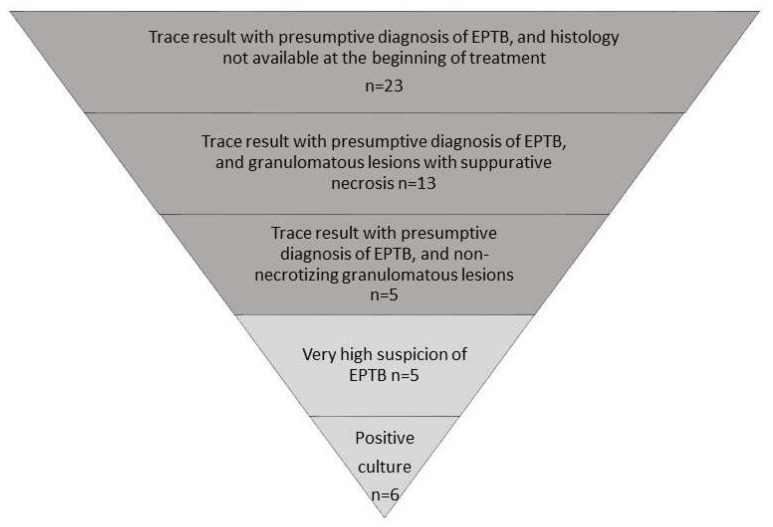
Role of the Ultra trace result in the decision to treat extrapulmonary tuberculosis (n = 52). EPTB: extra pulmonary tuberculosis. Presumptive diagnosis of EPTB: clinical and radiological findings consistent with EPTB.In dark grey, the number of patients for whom the decision to treat was influenced by the trace result. In light grey, the number of patients for whom the trace result did not influence the decision to treat.

An Ultra trace result with a presumptive diagnosis of TB led to the decision to treat 41/52 (78.8%) patients, without the histology result at the time of treatment for 23 (44.2%) patients, with granulomatous lesions and suppurative necrosis for 13 (25%) patients, and with non-necrotizing granulomatous lesions for 5 (9.6%) patients (Figure 2). The decision to treat was not influenced by the trace result in 11 cases: five were treated before diagnostic sampling on strong clinical and radiological suspicion (three pleural TB, two disseminated TB) and six patients were treated after the culture result (five lymph node TB, one extravertebral bone TB).

For 18/22 (81.8%) severe cases, treatment was started within 4 days after the trace result (13 the same day or the next day, 5 within 2 to 4 days).

All patients were started on first-line anti-TB drugs. Duration of treatment was available for 44 patients (ongoing treatment n = 3, patient died during the course of treatment n = 4, lost to follow-up n = 1). The median duration of treatment was 6.1 months. The duration of treatment was 9 to 11 months in 7 cases (disseminated n = 3, digestive n = 2, pleural n = 1, bone n = 1), and 12 months or more in 10 cases (disseminated n = 5, neuromeningeal n = 3, bone n = 1, lymph node n = 1).

### 3.5. Outcome

Among the 33 culture-positive patients, 22 had a favorable outcome, a 1-year-old child died of neuromeningeal TB, and 10 were lost to follow-up. Two patients had severe sequelae, (one shoulder TB requiring a shoulder prosthesis, one neuromeningeal TB with neurological sequelae).

Among the 13 culture-negative patients, 6 had a favorable outcome without any other associated diagnosis, 3 very old patients died (pleural forms n = 2, disseminated form n = 1), and 4 were lost to follow-up (epididymis, lymph node, pleural, and neuromeningeal forms).

Among the six patients for whom culture could not be performed, three had a favorable evolution without any other associated diagnosis and three were lost to follow-up (lymph node, pleural and disseminated forms with granulomatous lesions and suppurative necrosis).

## 4. Discussion

This study questions the influence of the Xpert MTB/RIF Ultra trace result in the treatment decision of EBTB. We were particularly interested in the severe and less frequent forms of EPTB, whose clinical signs may be nonspecific and lead to misdiagnosis. We analyzed 52 EPTB cases over a large part of the French territory. Treatment of severe forms was rapidly initiated after the trace result, without waiting for histology in almost half of the cases.

Early studies evaluating the Xpert Ultra test in areas of high TB prevalence, where HIV is endemic and there is a long history of TB, showed a high percentage of traces in sputum [10,11] up to 28% [10]. The authors proposed to reclassify these trace results as true negatives, especially in case of a history of treatment [10]. In a second phase, further Xpert Ultra trace studies were performed in low-endemic areas with patients suspected of having paucibacillary TB. Studies were conducted in areas with less treatment history and also fewer HIV-exposed patients. Mazzola et al. concluded that false positives results could be easily avoided by not performing the Ultra test on samples from patients with a clinically excluded diagnosis of TB or with a history of TB within 5 years [8]. According to Amedeo’s study, trace results in presumptive TB patients should be considered as true positives and treatment should be started promptly, except in cases of recent TB [9].

Our study was voluntarily limited to a strong clinical, radiological, and/or histological suspicion of EPTB without a history of tuberculosis. This is the first study including a significant number of severe forms; 20 (41.7%) adults had disseminated, digestive, or neuromeningeal forms of TB, and two children had a digestive or neuromeningeal form. The gold standard of studies remains culture, whereas a certain number of cases where TB was diagnosed and treated remained culture negative. The relatively high percentage of positive cultures in our series compared to that of Amedeo (71.7% versus 54.5%, respectively), may be due to the lower percentage of lymph node TB (55.9% versus 25%, respectively), that can remain sterile in culture. Severe neuromeningeal, digestive, and bone forms are paradoxically more often documented by culture in our study [9].

A precise analysis of histological results was realized by differentiating the presence of necrosis, which is highly suggestive of TB, from the absence of necrosis that can be observed in other diseases, such as sarcoidosis. A quarter of the EPTB cases were accompanied by granulomas without necrosis. This absence of necrosis should not delay treatment when the clinical and radiological signs are consistent with severe forms of TB.

Our study confirms the results of Aurilio’s study in Brazil [12] regarding whether the test was useful in children and adolescents; we identified two cases of pleural TB in adolescents, one case of neuromeningeal TB in a 1-year-old child that resulted in death, and one case of digestive TB in a 3-year-old child for whom a diagnosis of sarcoidosis had been made on non-necrotizing granulomatous lymphadenitis. Despite the limited number of pediatric patients, our study contributes to show the usefulness of the trace result given the difficult diagnosis of EPTB in children.

Amedeo’s study analyzed the role of trace result in the decision to treat. In this study, the decision to treat followed the trace result with a delay of 4 ± 4.7 days [9]. Among the patients with EPTB, 56% had lymph node forms, and 12% had bone forms. Our study confirmed that the trace result considerably shortened time before treatment because 87% of our severe forms were treated within 4 ± 3 days of the trace result, and 65% within 1 day.

Unfortunately, rifampicin resistance cannot be determined with a trace result, which is a major limitation in cases of high MDR-TB prevalence. The WHO recently recommended that, when individuals are being evaluated for extrapulmonary TB using cerebrospinal fluid, lymph nodes, and tissue specimens, MTBC detected trace result be considered as bacteriological confirmation of TB (i.e., a true positive result), and these patients should be treated with an appropriate regimen using first-line TB drugs unless the patients are at high risk of having MDR-TB [5]. Direct whole-genome sequencing (DWGS) of M tuberculosis enables the prediction of drug resistance either from bacterial strains or directly from smear-positive sputum samples [13,14]. DWGS has also been applied to smear-negative sputum samples with some success [14]. Since tests until now have been developed using sputa, it is unlikely that this test will be applied to extrapulmonary samples in the near future. However, pending future developments, a second Xpert Ultra test from another pulmonary (in mixed tuberculosis) or extrapulmonary sample can detect rifampicin resistance. In our study, nine patients had a second Xpert Ultra test positive on another sample with absence of rifampicin resistance available in six cases. This second rapid Xpert test did not delay the start of treatment and ensured the absence of MDR-TB, which is very useful in severe TB forms.

As limitations, this was a retrospective study, with subjects lost to follow-up.

In conclusion, the treatment of EPTB must be started as soon as possible, especially in the most severe forms, such as disseminated, digestive, or meningeal TB. Diagnosis can be based on the Xpert MTB/RIF Ultra test, even in the case of trace results. The test should be prescribed when there is a strong suspicion of EPTB without recent TB history as high pre-test probability limits the risk of a false positive result. If several samples are available, it may be useful to repeat the Xpert test. Clinicians should be informed about the higher risk of negative cultures with lymphadenopathy biopsies.

## Figures and Tables

**Table 1:** Characteristics of patients.

Characteristics of Patients	Number/Patients with Available Data n/N (%)
Male Sex	34/52 (65.4)
Median age (year, IQR)	30 (24–49)
Foreign-born residents	
Africa	30/52 (57.7)
Asia	6/52 (11.5)
East Europa	2/52 (3.8)
Metropolitan France	11/52 (21.2)
Other ^a^	3/52 (5.8)
HIV positive	2/49 (4.1)
Previous treated tuberculosis	2/50 (4.0)
Tuberculosis (TB) localization	
Lymph node ^b^	13/52 (25.0)
Disseminated ^c^	14/52 (26.9)
Bone ^d^	7/52 (13.5)
Pleural ^e^	9/52 (17.3)
Neuromeningeal ^f^	4/52 (7.7)
Digestive	4/52 (7.7)
Epididymis	1/52 (1.9)
Median treatment duration (months, IQR) ^g^ n = 44	6.1 (6–9.2)

IQR: Interquartile range. ^a^ Perou (n = 1), Guadeloupe (n = 1), Mauritius Island (n = 1). ^b^ Lymph node: pulmonary associated forms in four cases, pleural associated form in one case. ^c^ Disseminated TB: 1/isolation of *Mycobacterium tuberculosis* from blood, bone marrow, liver biopsy specimen, or > = 2 noncontiguous organs, 2/isolation of *M. tuberculosis* from one organ and histologic demonstration of TB from the bone marrow, liver biopsy, or another noncontiguous organ [2,3]. ^d^ Bone: vertebral TB n = 2, extra-vertebral TB n = 5. ^e^ Pleural: pulmonary associated form in one case and lymph node associated form in one case. ^f^ Neuromeningeal: pulmonary associated form in two cases, lymph node associated form in one case, uveitis associated form in one case. ^g^ eight patients excluded: died during treatment (n = 4), still on treatment (n = 3), lost to follow-up (n = 1).

**Table 2:** Culture analysis of samples with a trace result (N = 46).

Trace Sample Localization	Positive Cultures/Performed Cultures * n/N *	Mean Time between Trace Result and Culture, in Days (Extremes)
Lymph node	10/14	15.6
Bone tissue	8/9	22.5
Pleural tissue	3/3	16.3
Pleural fluid	1/5	17
Cerebrospinal fluid	3/4	19.3
Peritoneal tissue	7/8	16.6
Cardiac tissue	1/1	25.0
Epididymis tissue	0/1	ND
Cutaneous tissue	0/1	ND
Total	33/46 (71.7%)	18.9 (6–44)

ND not determined. * cultures were performed for 46 samples (4 lymph nodes, 1 pleural tissue, and 1 hepatic tissue embedded in paraffin were not cultured).

**Table 3:** Histology results.

Trace Sample *	Granulomatous Lesions with Suppurative Necrosis n/N	Non-Necrotizing Granulomatous Lesions n/N	Non-Specific Histology n/N
Lymph node	12/17	3/17	2/17
Bone tissue	4/8	3/8	1/8
Pleural tissue	3/4	1/4	0/4
Pleural fluid	0/4	1/4	3/4
Peritoneal tissue	4/7	2/7	1/7
Cerebrospinal fluid	0/2	0/2	2/2
Epididymis tissue	1/1	0/1	0/1
Cutaneous tissue	0/1	1/1	0/1
Hepatic tissue	1/1	0/1	0/1
Total of histology results *	25/45 (55.6%)	11/45 (24.4%)	9/45 (20.0%)

* Available histology results, n = 45/52 (86.5%).

## Data Availability

The data presented in this study are available on request from the corresponding author. The data are not publicly available due to privacy restrictions.
